# Cell Death Related Proteins Beyond Apoptosis in the CNS

**DOI:** 10.3389/fcell.2021.825747

**Published:** 2022-01-14

**Authors:** Bazhena Bahatyrevich-Kharitonik, Rafael Medina-Guzman, Alicia Flores-Cortes, Marta García-Cruzado, Edel Kavanagh, Miguel Angel Burguillos

**Affiliations:** Departamento de Bioquímica y Biología Molecular, Facultad de Farmacia, Universidad de Sevilla, and Instituto de Biomedicina de Sevilla (IBiS), Hospital Universitario Virgen del Rocío/CSIC, Seville, Spain

**Keywords:** caspase, Bcl-2, Bcl-xL, non-apoptotic, CNS, sexual dymorphism, neurodegeneration

## Abstract

Cell death related (CDR) proteins are a diverse group of proteins whose original function was ascribed to apoptotic cell death signaling. Recently, descriptions of non-apoptotic functions for CDR proteins have increased. In this minireview, we comment on recent studies of CDR proteins outside the field of apoptosis in the CNS, encompassing areas such as the inflammasome and non-apoptotic cell death, cytoskeleton reorganization, synaptic plasticity, mitophagy, neurodegeneration and calcium signaling among others. Furthermore, we discuss the evolution of proteomic techniques used to predict caspase substrates that could potentially explain their non-apoptotic roles. Finally, we address new concepts in the field of non-apoptotic functions of CDR proteins that require further research such the effect of sexual dimorphism on non-apoptotic CDR protein function and the emergence of zymogen-specific caspase functions.

## Introduction

CDR proteins include a variety of proteins (e.g. caspases, members of the Bcl-2 family, etc.) whose actions directly affect the outcome of the apoptotic cell death (i.e. pro-apoptotic vs. anti-apoptotic proteins). Considerable progress had been made in the field of apoptosis since its seminal discovery by the labs of Brenner, Sulston and Horvitz, whose findings were recognized with the Nobel Prize in Physiology or Medicine in 2002. Since then, knowledge of the individual proteins critical to the apoptotic process has increased considerably, and in fact widened to encompass new and exciting non-apoptotic functions. In this mini-review, we examine the non-apoptotic roles of caspases as well as other CDR proteins such as members of the Bcl-2 protein family and X-chromosome-linked inhibitor of apoptosis (XIAP). We will focus on particularly in the CNS, where these non-apoptotic roles play great relevance in the physiology of the different cell types forming it. We will briefly summarize these non-apoptotic functions [which have already been reviewed in depth in the CNS (Shen et al., 2018; Espinosa-Oliva et al., 2019; Rodríguez-Gómez et al., 2020)] to focus more on the most recent findings in this exciting field.

### Diversity of Caspase Functions

Caspases are a family of cysteine proteases (Schauperl et al., 2015; Julien and Wells, 2017), traditionally known for their roles in apoptotic cell death during development and disease (Shen et al., 2018). However, since the late nineties, seminal studies have described various non-apoptotic roles for caspases, such as the differentiation of lens fiber cells (Ishizaki et al., 1998), and processes necessary for erythropoiesis (De Maria et al., 1999), erythroid terminal differentiation (Zermati et al., 2001) and maturation (Carlile et al., 2004). Since then, the list of distinct caspase functions has increased and diversified significantly, including within the CNS.

### Inflammasome, Pyroptosis and Necroptosis

Some of the first non-apoptotic functions described for caspases were linked to inflammatory processes, and alternative cell death pathways, pyroptosis and necroptosis. The inflammasome is a multiprotein intracellular complex (Martinon et al., 2002), triggered by external stimuli that leads to the production and release of pro-inflammatory cytokines IL-1β and IL-18. The complex consists of a sensor protein such as NLRP1 or NLRP3, an adaptor protein named apoptosis-associated speck-like protein containing a caspase activation and recruitment domains (ASC), and caspase-1, caspase-11 (Schroder and Tschopp, 2010; Yi, 2018; Chen et al., 2021) or caspase-8 (Zhang et al., 2018) in microglia cells. There is a study that suggests that caspase-6, implicated in axonal degeneration and cognitive decline in AD (Simon et al., 2012; Geden et al., 2019), may play an additional role in inflammasome activation in neurons (Kaushal et al., 2015). Although the mechanism is not well elucidated, the authors showed that NLRP1-Caspase-1 activation of caspase-6 in neurons caused neurodegeneration in hippocampal regions CA3 and DG in an AD model (Kaushal et al., 2015), implicating this pathway as a therapeutic target (Flores et al., 2021).

Inflammasome activation may progress to a regulated cell death process termed pyroptosis, during which caspase-1 cleaves gasdermin D (GSDMD) provoking plasma membrane pore formation that causes cell lysis (Shi et al., 2015). Recently, studies have linked apoptotic cell death pathways with pyroptosis in the CNS, specifically in human primary microglia (McKenzie et al., 2020a). McKenzie et al. illustrated that active caspase-3/7 may mediate pyroptosis *via* ROCK1, alongside the well described caspase-1-GSDMD pathway, in microglia in multiple sclerosis and its animal model experimental autoimmune encephalomyelitis. Of interest, in human microglia, caspase-3/7 activation during pyroptosis required inflammasome activation, and treatment with VX-765 (caspase-1 inhibitor) reduced cleaved caspase-3 under pyroptotic but not apoptotic stimulus. This contribution supports the idea of convergence of different cell death pathways in the CNS and provides a novel therapeutic target for pyroptotic mediated cell death (McKenzie et al., 2020b).

Necroptosis in microglia is a regulated cell death mechanism initiated by the interaction of receptor-interacting protein kinase (RIPK)-1 and RIPK3, mixed lineage kinase domain-like protein (MKLK) and caspase-8 (Tait et al., 2014; Ofengeim et al., 2015). This interaction occurs upon ligand binding to death receptors such as TNFR or FAS. Under these conditions, activation of caspase-8 promotes apoptosis and actively inhibits necroptosis by direct cleavage of RIPK1 and RIPK3. By contrast, in the absence of caspase-8 following genetic ablation, pharmacological inhibition, or as a result of certain viral infections, RIPK1 and RIPK3 are stabilized and recruit mixed-lineage kinase domain-like protein (MLKL) into complex IIb, also known as the necrosome, which initiates necroptosis (Zhang et al., 2017).

### Caspases and Aberrant Proteostasis

There are common pathological features shared by various neurodegenerative diseases such as Huntington’s disease, Alzheimer’s disease (AD), Parkinson’s disease, and frontotemporal lobar degeneration. One is a chronic inflammatory response and another is an aberrant proteostasis which leads to aggregation and deposition of intracellular and/or extracellular protein inclusions (Lee et al., 2011). Many of these neurodegeneration-linked aggregation-prone proteins such as amyloid precursor protein (APP) (Gervais et al., 1999; Park et al., 2020), tau (Cotman et al., 2005; de Calignon et al., 2010), α-synuclein (Ma et al., 2018), or huntingtin (Wellington et al., 2000; Martin et al., 2019) can be processed by caspases, generating toxic fragments that aggregate, thereby linking caspase activity with the etiopathogenesis of these diseases. Recent studies have described new characteristics of these caspase-generated toxic fragments. One example in mouse primary cortical neurons is a caspase-3 generated tau fragment, that aggregates and forms neurofibrillary tangles and induces a reduction of Ca2+ levels in the endoplasmic reticulum (ER) and enhancement of ER-mitochondria communication (Cieri et al., 2018). The exact relationship between caspase-3-cleaved tau and the enhancement of the contact sites between ER and mitochondria, and Ca2+ levels is unknown. However, the authors suggest that this could be an important pathological event in tau-related neurodegenerative disease. Similarly, the ability to impact neuronal ER-mitochondria communication, has been reported for α-synuclein (Calì et al., 2012), and β-amyloid (Aβ) oligomers (Calvo-Rodriguez et al., 2019). While the role for caspase-3 in facilitating nucleation-dependent tau filament formation is well described, the importance of caspase-6 in this process is not so evident. Early studies described caspase-6 as capable of processing tau (Horowitz et al., 2004; Rissman et al., 2004). However, in hippocampal and cortical neurons tau fragments generated by caspase-6 are insufficient *per se* to induce tau pathogenesis, as shown recently using a transgenic knock-in mouse model expressing full-length human tau together with human Casp6 (Noël et al., 2021).

Altered functional connectivity and synaptic degeneration is the best pathological correlate of the cognitive decline seen in AD that occurs at the early stages of disease (Terry et al., 1991). The role of caspase-3 in this process, based on its ability to cleave APP and release cytotoxic fragments, has been highlighted several times (D’Amelio et al., 2011; Louneva et al., 2008; Lu et al., 2000; Banwait et al., 2008). However, the intricate relationship amongst caspase-cleaved APP fragments, caspase activation and synaptic loss has not been directly shown. Recent experiments in hippocampal neuronal organotypic slice cultures from APP knock-in mice that are resistant to cleavage by caspase-3 prevented Aβ-dependent caspase activation and release of the putatively cytotoxic C31 peptide, which plays an essential role in Aβ-induced dendritic spine loss and impairment in synaptic plasticity (Park et al., 2020).

Synaptic plasticity in the context of learning and memory as well as neurodegeneration and neuronal injury can also be affected by caspase activity. To date, involvement of pro-apoptotic proteins (Li et al., 2010; Jiao and Li, 2011) such as caspase-3, Bad, and Bax have been shown to regulate internalization of α-amino-3-hydroxy-5-methyl-4-isoxazolepropionic acid receptor (AMPAR) subunits in hippocampal synapses, a process necessary for long-term depression (LTD) (D’Amelio et al., 2012). Additionally, a recent Parkinson’s disease study reported that in striatal medium spiny neurons present in corticostriatal slices from PINK1 knockout (KO) mice exhibit impaired LTD, which was restored with low doses of caspase-3 activator α-(Trichloromethyl)-4-pyridineethanol. Interestingly when LTD was induced in PINK1 KO mice, reduced activity of caspase-3 compared to wild type was observed (Imbriani et al., 2019). It is likely the PINK1 deficiency impacts the fine modulation of caspase cascade which in turn may perturb long-term synaptic plasticity machinery, leading to synaptic dysfunction. Peripheral nerve injury downregulates caspase-3 expression in neurons present in the anterior cingulate cortex, where its interaction with AMPAR regulates LTD. The absence of LTD, due to downregulation of caspase-3, enhances synaptic transmission in somatosensory pathways and contributes to peripheral hypersensitivity. Intriguingly, caspase-3 directly regulates LTD *via* AMPAR subunit cleavage and subsequent internalization (Wang et al., 2020).

### Caspase-Mediated Reorganization of the Cytoskeleton

Caspases are involved in the reorganization of the cytoskeleton of neurons, either for the correct wiring of the olfactory sensory neurons axons (Ohsawa et al., 2010) or by eliminating the dendrites, dendritic spines or axons (Cusack et al., 2013; Kaushal et al., 2015). Axonal or dendritic pruning allows for selective degeneration of excessive, misguided, or unnecessary neuronal extensions without degeneration of the entire cell, and occurs not only during brain development but also for plasticity in the mature brain (Saxena and Caroni, 2007). Importantly, failure of this process has been associated with neurodevelopmental (Riccomagno and Kolodkin, 2015; Thomas et al., 2016), and neurodegenerative disorders (Burke and O'Malley, 2013; Stokin et al., 2005; Vickers et al., 2009). Controlled axonal pruning is similar in many ways to the active self-destruction of cells during apoptosis (Geden et al., 2019). Hertz et al. found in Tropomyosin receptor kinase A positive neurons that dephosphorylation and cleavage of RUFY3, an adaptor protein for small GTPases (Kitagishi and Matsuda, 2013), which acts downstream of or in parallel to caspase 3 activation, is required for TRKA+ sensory axon degeneration upon trophic deprivation. Importantly, depletion of RUFY3 protects from axonal degeneration even in presence of active caspase-3 (Hertz et al., 2019). This protein could be a possible new checkpoint, allowing neurons to locally control caspase-driven degeneration. It is noteworthy that RUFY3 expression was increased in the olfactory bulbs of AD patients compared with healthy individuals across early and advanced AD (Zelaya et al., 2015).

Caspase-3 can control dendritic spine density and architecture (Mukherjee and Williams, 2017). Under stress conditions caused by oligomycin A in primary cultured hippocampal neurons, mitochondrial F1Fo ATP synthase dysfunction induces non-apoptotic caspase-3 activation in dendrites concurrent with dendritic spine retraction and spinogenesis suppression. Moreover, the inhibition of caspase activation using a pan-caspase inhibitor protected against dendritic spine damage. This finding illustrates the contribution of non-apoptotic caspase activity to dendritic spine turnover (Chen et al., 2020).

### Non-Apoptotic Functions of Other CDR Proteins

CNS non-apoptotic functions have been described for CDR proteins such as XIAP and Bcl-2 family. In some cases, CDR proteins modulate caspase activity directly, affecting non-apoptotic caspase functions. For instance, regulation of XIAP degradation via ubiquitination may be responsible for some of the non-apoptotic roles of caspase-3. Specifically, XIAP inhibits the ability of caspase-3 to shape dendrites through cleavage of microtubules, a process that, unregulated, has been observed in various autism spectrum disorders (Khatri et al., 2018). Furthermore, in hippocampal neurons XIAP modulates caspase-3 mediated AMPAR internalization in synapses in NMDA receptor-dependent LTD (Coccia et al., 2020). The pro-apoptotic proteins Bax and Bak trigger non-apoptotic caspase activity required for synaptic rearrangement between corticospinal neurons and muscles in mice during early development (Gu et al., 2017).

Non-caspase CDR proteins also contribute to non-apoptotic CNS processes independently of their regulation of caspase activity. One example is the control of mitochondrial Ca2+ homeostasis through direct interaction of BCL-2 family members with Ca2+ transporters (such as IP3Rs) present at mitochondria-ER contact points. Bcl-2, Bcl-xL and Mcl-1 interact with IP3Rs (Nougarede et al., 2018). The outcome of this interaction on the permeability of IP3Rs is not yet clear. For instance, in the case of Bcl-2 homolog Nrh/BCL2L10, its interaction with I3PRs negatively regulates ER-Ca2+ release (Nougarede et al., 2018). In the case of Bcl-xL, it’s a matter of debate whether it’s interaction with IP3Rs affects IP3R permeability (topic reviewed in (Popgeorgiev and Gillet, 2021)). It has been reported that Bcl-xL interaction with IP3Rs can promote calcium release and regulate mitochondrial metabolism, due to the requirement of calcium by several enzymes of the tri-carboxylic acid cycle and the electronic transport chain, as well as the ADP/ATP translocator and ATP synthase in neurons (Bas et al., 2021). However a recent study has shown that Bcl-xL inhibits IP3R-mediated Ca2+ release, contributing to the anti-apoptotic response against Ca2+-driven apoptosis induced by drugs such as Staurosporine (Rosa et al., 2021). Bcl-xL can indirectly regulate cellular energy levels by modulating ADP/ATP trafficking between mitochondria and the cytoplasm, through its interaction with voltage-dependent anion channels (VDAC) (Tan and Colombini, 2007). Furthermore in neurons, Bcl-xL directly interacts with the ATP synthase β subunit (Chen et al., 2011), stabilizing the membrane potential across the inner mitochondrial membrane and increasing overall energetic efficiency. The interaction between Bcl-xL and the ATP synthase β-subunit is blocked upon Bcl-xL phosphorylation by the cyclin B1-Cdk1 complex, a process that is dysregulated in AD neurons (Veas-Pérez de Tudela et al., 2015). One of the consequences of an increase in neuronal ATP is the promotion of synaptic release and uptake of neurotransmitters. Interestingly, Bcl-xL itself promotes endocytic vesicle retrieval in hippocampal neurons through protein–protein interaction with components of the clathrin complex (Li et al., 2013). Bcl-xL affects mitochondrial dynamics, in particular mitochondria fission, by interacting with dynamin-related protein 1 in cultured hippocampal neurons (Li et al., 2008). Additionally, Bcl-xL inhibits mitophagy either *via* its inhibitory interaction with Beclin-1 [reviewed in Zhou et al. (2011)] or by blocking Parkin translocation to depolarized mitochondria (Hollville et al., 2014).

### Proteomic Search for Non-Apoptotic Caspase Substrates in the CNS

New non-apoptotic roles are constantly emerging for the CDR proteins. The involvement of caspase enzyme activity in such a wide range of key biological signaling processes has led to a strong interest in the identification of caspase substrates. Key to the detection of caspase substrates has been the resolution of the caspase tetrapeptide substrate recognition motif, which specifies the preferred amino acid sequence recognized by specific caspases. Although the motifs have in common an aspartate (D) at position P1 (Talanian et al., 1997), the amino acids at the other positions vary for most caspases, and are merely a predictor of caspase cleavage site. For example, only 1% of known caspase-3/7 substrates contain the full DEVD* substrate recognition motif (Julien and Wells, 2017). Additionally, it should also be noted that many substrates are targets for multiple caspases, caspase-3 being the most promiscuous of the family (Benkova et al., 2009). Bioinformatic tools have been developed to identify potential caspase substrates using algorithms that predict substrate recognition motifs within a protein’s amino acid sequence such as PEPS (Lohmüller et al., 2003), GraBCas (Backes et al., 2005), and CasCleave, with the latter taking into account protein structural information and solvent accessibility (Song et al., 2010).

Experimentally, the hunt for caspase substrates at the proteomic level in native cells involves incubating a recombinant caspase with cell lysate, isolating the subsequently cleaved proteins with newly exposed N-termini, and digesting and identifying the peptide fragments by mass spectrometry (Gevaert et al., 2003; Mahrus et al., 2008). A variation of this method provides more certainty of the responsible caspase by neutralizing proteases in the cell lysate prior to the addition of exogenous purified caspase (Julien et al., 2016). These methods have resulted in the identification of hundreds of native substrates for many caspases (Benkova et al., 2009) under apoptotic (Dix et al., 2008; Mahrus et al., 2008) and inflammatory conditions (Agard et al., 2010) in Jurkat T lymphocytes or THP-1 monocyte cell lines (Araya et al., 2021). The search for CNS-relevant or CNS specific caspase substrates has been less well studied. Recently, once such study carried out in isolated neuronal synaptosomes from C57 mice identified approximately 70 substrates, 48 previously unidentified, of caspases 3 and 6 (Victor et al., 2018), signaling the importance of caspase activity in synaptic plasticity. Similarly, in a model of auditory brainstem development, extracellular vesicles were isolated from the brainstems of embryonic chicks, and subjected to nanoLC-MS/MS, leading to the identification of caspase-3 substrates (288/5,653 unique proteins), of which 193 were novel (Weghorst et al., 2020). Based on a functional enrichment analysis of the novel caspase-3 substrates found, the authors suggest a non-apoptotic role of caspase-3 in axon guidance and maturation of auditory nuclei. These proteomic studies highlight the utility of non-biased approaches to caspase substrate mining.

**FIGURE 1:**
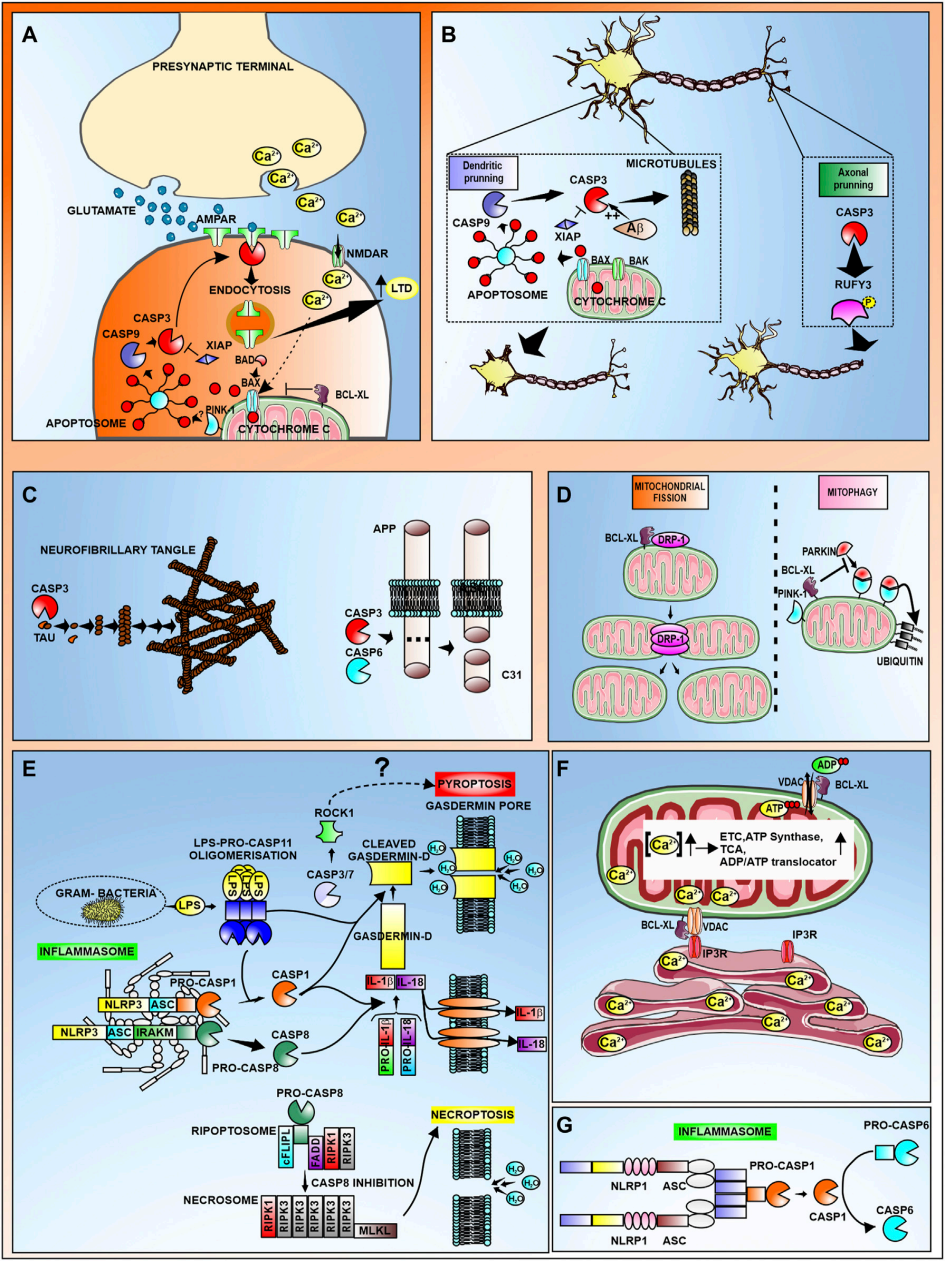
Schematic representation the non-apoptotic roles of CDR proteins in CNS. **(A)** Induction of Long term depression (LTD) requires active caspase-3 mediated internalization of AMPAR subunits. Calcium released at the presynaptic terminal is transported through NMDAR, promoting cytochrome c release from mitochondria. Cytochrome c can also be released *via* Bad/Bax translocation. Subsequent formation of the apoptosome leads to activation of caspase-3 at the postsynaptic terminal, where the active form binds to AMPAR, promoting its endocytosis. Internalization of AMPAR results in increased LTD. This process can be antagonized by the proteins XIAP and Bcl-xL. **(B)** Caspase-3 mediates dendritic and axonal pruning *via* cytoskeletal destabilization. During dendritic pruning, caspase-3 cleaves microtubules leading to a reduction in dendritic spines. In AD, Aβ promotes caspase-3 activation. Active caspase-3 cleaves dephosphorylated RUFY3 to promote axonal pruning **(C)** Among the neuronal substrates of caspases are aggregation-prone proteins such as Tau and APP. **(D)** Bcl-xL promotes mitochondrial fission by interacting with DRP-1, and inhibits mitophagy by preventing the Parkin-PINK1 ubiquitination of mitochondrial proteins. **(E)** Representation of canonical (caspase-1) and non-canonical (caspase-11 or capase-8) inflammasome activation that leads to the processing and release of IL-1β and IL-18 and the processing of Gasdermin D leading to pyroptosis. It has been suggested that active caspase-3/7 can also mediate pyroptosis through cleavage of ROCK-1. The complex formed by mixed lineage kinase domain like pseudokinase (MLKL) together with Receptor-interacting protein kinase (RIPK)-1 and RIPK3, can promote necroptosis in the absence of active caspase-8. **(F)** Bcl-xL promotes Calcium trafficking from the ER to mitochondria through interaction with IP3R and VDAC. The resulting increase in mitochondrial calcium concentration activates several proteins in the electron transport chain (ETC), tricarboxylic acid cycle (TCA), as well as ATP synthase and the ADP/ATP translocator which altogether leads to the increase in ATP production. Also, Bcl-xL modulates ADP/ATP trafficking from the cytosol to the mitochondria through its interaction with VDAC. **(G)** Activation of caspase-6 mediated by the NLRP1-caspase-1 inflammasome in neurons has been implicated in hippocampal AD neurodegeneration.

We have included a table summarizing the different non-apoptotic roles of the different CDRs discussed in this manuscript (Table 1) as well as a schematic representation for some of those non-apoptotic roles of CDR proteins in CNS (Figure 1).

**TABLE 1:** CNS functions of CDR proteins.

CDR proteins	Role	CNS cell type	References
Bad, Bax	Participate together with cleaved caspase-3 in AMPAR internalization during LTD	Hippocampal neurons	D'Amelio et al. (2011), D'Amelio et al. (2012)
	Mediate the synaptic rearrangement between cortical neurons and muscles	Corticospinal Neurons	Gu et al. (2017)
Bcl-2, Bcl-xL, Mcl-1	Interact with IP3Rs promoting ER calcium release towards mitochondria. Released Calcium controls TCA enzyme activities	Neurons	Li et al. (2008), Bas et al. (2021)
Bcl-xL	Interacts with VDAC mediating ADP/ATP trafficking between mitochondria and cytoplasm	Neurons	Tan and Colombini, (2007)
	Interacts with ATP synthase β subunit, promoting energy efficiency in mitochondria. Indirectly promotes synaptic release and uptake of neurotransmitters	Neurons	Chen et al. (2011)
	Interacts with DRP-1 to promote mitochondria fission	Neurons	Li et al. (2008)
	Interacts with Beclin-1 or Parkin to inhibit mitophagy	Neurons	Zhou et al. (2011), Hollville et al. (2014)
Caspase-1	Initiator caspase responsible for the canonical inflammasome pathway, pyroptosis (*via* GSDMD cleavage) or initiation (with NLRP1) of caspase-6-dependent neurodegeneration	Microglia, Neurons	Kaushal et al. (2015), Shi et al. (2015), Chen et al. (2021)
	Processes endogenous α-synuclein	Neurons	Ma et al. (2018)
	Processes mutant huntingtin at position D572 promoting Huntington’s disease pathogenesis	Neurons	Martin et al. (2019)
Caspase-3	Mediates pyroptosis by cooperating with GSDMD *via* ROCK1 cleavage in Multiple Sclerosis and autoimmune encephalomyelitis	Microglia	McKenzie et al. (2020a), McKenzie et al. (2020b)
	Cleaves APP to the cytotoxic C31 peptide which plays a role in spine loss and impairment of synaptic plasticity	Neurons	Gervais et al. (1999), Park et al. (2020), D'Amelio et al. (2011), Louneva et al. (2008), Lu et al. (2000)
	Cleaves Tau which reduces ER Calcium	Neurons	Cotman et al. (2005), de Calignon et al. (2010), Cieri et al. (2018)
	Cleaves mutant huntingtin	Neurons	Wellington et al. (2000)
	Interacts with AMPAR to promote AMPAR internalization during LTD.	Hippocampal neurons	D'Amelio et al. (2012), Imbriani et al. (2019)
	Cleaves RUFY3 to promote axonal degeneration	TRKA+ neurons	Hertz et al. (2019)
	Promotes dendritic spine retraction and inhibits spinogenesis	Neurons	Chen et al. (2020)
	Procaspase-3 promotes mitochondrial biogenesis	Dopaminergic neurons	Kim et al. (2018)
Caspase-6	Processed by NLRP1-caspase-1, promotes CA3 and DG neurodegeneration in an AD mouse model	Neurons	Kaushal et al. (2015), Flores et al. (2021)
	Proteolysis of the β-secretase site of APP	Neurons	Gervais et al. (1999)
	Cleaves Tau at various sites	Neurons	Horowitz et al. (2004), Rissman et al. (2004), Cotman et al. (2005), de Calignon et al. (2010), Noël et al. (2021)
	Cleaves Htt protein	Neurons	Wellington et al. (2000), Martin et al. (2019)
Caspase-7	Mediates pyroptosis by cooperating with GSDMD *via* ROCK1 cleavage in Multiple Sclerosis and autoimmune encephalomyelitis	Microglia	McKenzie et al. (2020a), McKenzie et al. (2020b)
	Cleavage of Tau	Neurons	de Calignon et al. (2010)
Caspase-8	Promotes IL-1β release as part of a noncanonical inflammasome	Microglia	Zhang et al. (2018)
	Lack of caspase-8 activity induces necroptosis *via* recruitment and stabilization of RIPK1/3 and MLKL upon TNF-α or Fas ligand interaction	Microglia	Tait et al. (2014), Ofengeim et al. (2015), Zhang et al. (2017)
Caspase-11	Initiator caspase responsible for the no-canonical inflammasome pathway and pyroptosis (*via* GSDMD cleavage)	Microglia	Yi (2018)
XIAP	Inhibits caspase-3 dendritic remodelling and prevents caspase-3 AMPAR internalization	Neurons	Khatri et al. (2018), Coccia et al. (2020)

### Sexual Dimorphism in CDR Protein Function

In the CNS, as well as in the periphery, there is increasing evidence of a gender-dependent natural bias in various disorders. In AD and Parkinson’s disease, the ratio of affected males to females and the severity of the disease depending on gender, differs significantly (Ullah et al., 2019). In ischemic stroke, females suffer worse cognitive outcomes than males (Selvamani and Sohrabji, 2017; Raval et al., 2019). An example of this dichotomy can be found in stroke, where mechanisms underlying ischemic cell death are caspase-independent in males and caspase-dependent in females (Siegel et al., 2011). The explanation for this difference in cell death mechanisms remains elusive although some studies have found differences in miRNAs expressed in each gender upon injury that could be linked with the exertion of one or other cell death mechanism. In an ischemic mouse model, the authors found an increase in the levels of miR-23a that targets XIAP in females, promoting caspase-dependent cell death mechanisms in this gender (Siegel et al., 2011). In a separate study, high levels of miR363-3p, that targets caspase-3, were found in the serum of adult female rats with small infarct volume compared to other groups with greater stroke-associated impairment (Selvamani and Sohrabji, 2017). Could sexual dimorphism affecting cell death mechanisms also affect non-apoptotic caspase roles? There are some indications that support this hypothesis. For instance, in a glaucoma model, treatment with estradiol inhibited the ability of caspase-3 to process tau, decreasing the formation of neurofibrillary tangles (Means et al., 2021). In a separate study, an increase in the expression of inflammasome components (including caspase-1) in aged female rats compared to aged-match males was linked to their decrease in estrogen levels (Raval et al., 2019). Taken together, these results suggest that the inclusion of both genders in future studies of non-apoptotic roles for caspase and other CDR proteins should be taken into consideration.

## Discussion

A combination of traditional techniques in biochemistry, molecular biology and microscopy with other multi-omic and bioinformatic approaches have made possible the discovery of new non-apoptotic roles for caspase and other CDR proteins. Many of these new non apoptotic roles are based on well-established mechanisms, like for instance caspase’s enzymatic activity, that depending on the substrate will play either an apoptotic or non-apoptotic role. Interestingly, a new study highlighting a non-apoptotic function of caspases independently of their enzymatic activity has recently emerged in the CNS. Studies performed by Kim and colleagues have described a novel role for procaspase-3 in promoting mitochondrial biogenesis in dopaminergic neurons through induction of the synthesis of the transcription factors Tfam and Nrf-1, necessary for mitochondrial biogenesis (Kim et al., 2018). This highlights the possibility of new functions for other caspases independently of their protease activity. These roles could also be affected by a potential gender dependent natural bias and should be taken into consideration in future studies for non-apoptotic roles for CDR proteins.

## Abbreviations

AMPAR, α-amino-3-hydroxy-5-methyl-4-isoxazolepropionic acid receptor; APP, amyloid precursor protein; CDR, Cell Death Related; GSDMD, Gasdermin D; LTD, long term depression; RIPK, Receptor interacting protein kinase; VDAC, voltage-dependent anion channels.
